# Supplementary material for: Chromothripsis during telomere crisis is independent of NHEJ, and consistent with a replicative origin
Source: Genome Res. 2019 May;29(5):737–49. doi: 10.1101/gr.240705.118 (PMC6499312; doi:10.1101/gr.240705.118)
Supplement: Supplemental Material [file supp_gr.240705.118_Supplemental_file_1.zip › contigs/annotated_contigs/DB50/contig.4.DB50_length_1049_mean_cov_7.68732125834.docx]

**DB50_length_1049_mean_cov_7.68732125834**

```
GAGAGATGAGAGAAACAACGACTGGAGGGATTAAGAAGAACTCTTAGGAGCAGCTTGGCCTAAAGGGTAAGAGTTGTGTGCCTTAAGTC
 >chr5:79287169-79287676 + E=6e-287
AGACCCACCAGTAATTCTTCAAATATTAATATTTATTAGATCCCTAGAGTGTTCCAAACCCTTTGAACATTAAATCTGTGTGATGCCTA

AATTAACCTGTTTGTAAAACAGGGATGACCCAGTATTTACAAGATTGTGGTATTTACAATGTCGGAGAGAAAGAACTTGGCACACAGGA

GGCACTCAGTAATTGTTCAACCCCTTCTCCCATGTATGTGGCCTCTGCCTGCCTTTCCAGCCTCAACCTGTTCTCACCCTCATGTTCAC

TGGCACAACCCTGCTTAAAACTTGCAGTATTTCTGAACGTCAGGTAAATTGTGCCCACCTTCAGTGCTGGTCCTTCAATCTGGAATATC

TTTCTTCCCTATGCAGCTGCTCCCTCAAAAATGAGCTTAACTCACCTTCCCTGTGAT|GTTTT|AGTTAAGAAGATCCGACCATCTCAG
                                                          >chr5:79287877-79288199 - E=1
TGGAATAGAGGAAAGAACTCAGAGGCATGACTGCGAGGGTGTCCCAGAAAGACTACTGTGCCTTCTAGAAAAAAGACCCAGCACCCAAG
e-181
TTCTAACTCTGAGACCCAGTCATATGTTTGGATCATAGCATTTCCAAGACCAAAATTTCCTCTTTAATAGTTTTTCTGAGAAACTGTCA

GGTTTAACGTCAAGAAACATTTAGTCTGTGCTCATTAGGCCCACAGACATGACGAGCTACATGATTTGAGGGGCCCAGTCCAAAATAAA

AATGCAGAGCCCCTTGTTC|AAAACT|CAAAAGAAACCTCTGCAACTTTTCGTAAAATTACTATTCAACAAATATTTGCTAGGCACTCC
                    >chr5:79288194-79288363 + E=1e-89
CTTACTGTTTACATAATCATTCAAATTACAGTGCCAGCCTGCTAAAGTCTTTTTTACATGTTTCAAAACCAAGAGTTCTAAGAAATGAA

AAAGAGATATG|T|GCACAAACAGGCTGAATACTATTCCAGAGAACCACACAGAAAATTGAAGCCTTCAGAAATTA            >chr5:79289180-79289243 + E=6e-23
```
